# Imaging the carotid atherosclerotic plaque

**DOI:** 10.1530/VB-19-0010

**Published:** 2019-06-28

**Authors:** Sandra Neumann, Elena G Milano, Chiara Bucciarelli-Ducci, Giovanni Biglino

**Affiliations:** 1 Research and Imaging Centre (CRIC) Bristol, University of Bristol, Bristol, UK; 2 UCL Institute of Cardiovascular Science and Great Ormond Street Hospital for Children, London, UK; 3 Department of Surgery, Dentistry, Paediatrics and Gynaecology, University of Verona, Verona, Italy; 4 University Hospitals Bristol, NHS Foundation Trust, Bristol, UK; 5 Bristol Medical School, University of Bristol, Bristol, UK

**Keywords:** imaging, cardiology, vascular disease

## Abstract

This mini review provides a concise overview of imaging techniques that are currently used to image the atheroscletoric plaque in the carotid artery *in vivo*. The main techniques include ultrasound imaging, X-ray imaging, magnetic resonance imaging and positron emission tomography imaging. Each technique has advantages and limitations and may be chosen depending on the availability, cost and clinical justification for its use. Common to all the imaging techniques presented here is the need for a skilled imaging professional to allow for high reliability and repeatability. While ultrasound-based imaging currently is regarded as a first line technique in clinical practice, the use of other techniques such as computed tomography angiography or magnetic resonance angiography need to be considered in the presence of significant stenosis with or without symptoms. Advancements in these two modalities, as well as in positron emission tomography imaging, are increasingly moving toward a better understanding of the risk-stratification and pre-interventional monitoring of patients at risk of plaque rupture as well as early identification of plaque development and better understanding of plaque composition (e.g. metabolic imaging).

## Introduction

**Figure 1:**
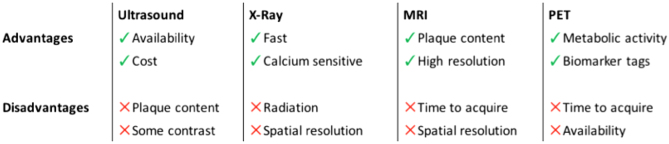
Summary of imaging techniques and relative advantages. MRI, magnetic resonance imaging; PET, positron emission tomography.

The study of the atherosclerotic plaque is of great interest for screening and assessment of patients at risk of cerebrovascular accidents (1). Several non-invasive imaging techniques can be used to study the atherosclerotic plaque. The plaque is typically composed of macrophage cells, fatty residue, calcium and fibrous connective tissue and debris, causing a narrowing of the vessel lumen. The technique and modality chosen should be optimized for the study in question. This mini review aims to provide an overview of the techniques used to image non-invasively the carotid plaque *in vivo*. A summary of the techniques discussed is shown in Fig. 1.

## Ultrasound-based imaging

**Figure 2:**
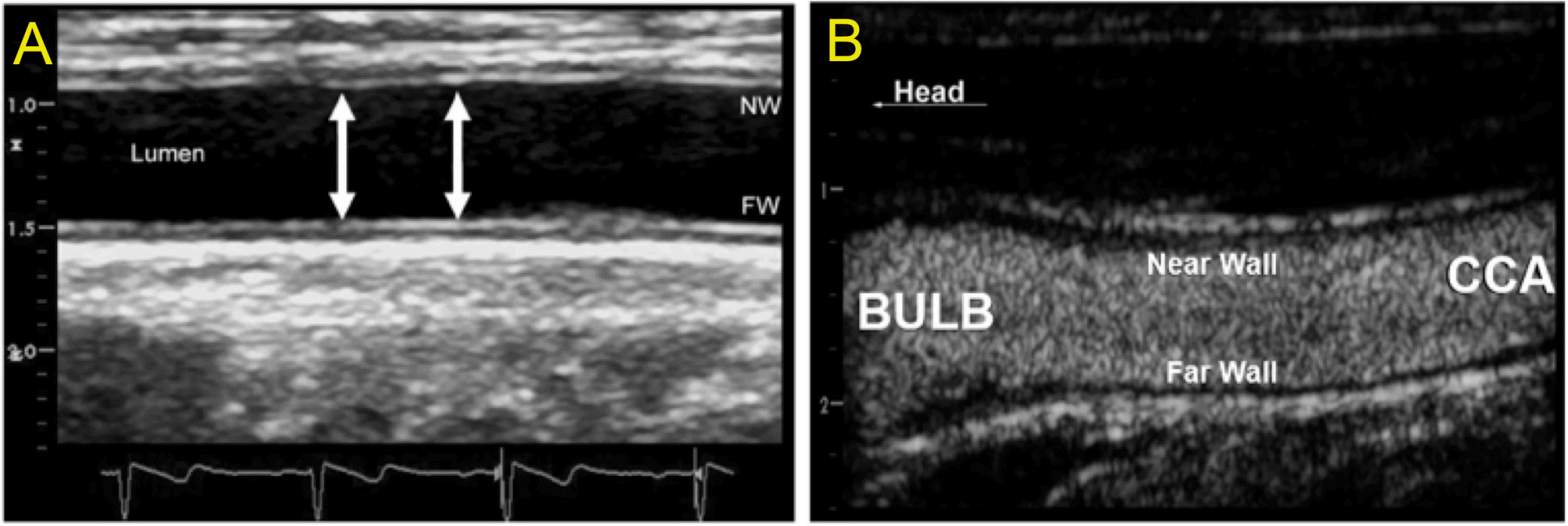
(A) Example of ultrasound-acquired images of the common carotid with B-mode non-contrast-enhanced ultrasonography and visualization of intima-media thickness in the near wall (NW) and far wall (FW); (B) example of near wall and far wall visualization using contrast‐enhanced ultrasound imaging. Reproduced from Shah BN, Chahal NS, Kooner JS & Senior R; Contrast‐enhanced ultrasonography vs B‐mode ultrasound for visualization of intima‐media thickness and detection of plaques in human carotid arteries; *Echocardiography* 2017, volume **34**, pages 723–730 (33). Copyright 2017 John Wiley and Sons.

Ultrasound-based imaging has the advantages of being non-invasive, radiation free, not requiring contrast medium and associated to only minimal discomfort to the patient. The technique is cost-effective, widely available and allows both the visualization and the grading of the atherosclerotic plaque severity. Examples of ultrasound imaging are shown in Fig. 2.

### Carotid intima-media thickness

Carotid intima-media thickness (CIMT) imaging uses a linear array transducer with a frequency of at least 7 MHz in B-mode (2, 3). Lower frequencies are not sufficient to obtain near-field resolution for the imaging of superficial vessels such as the carotid artery. The transducer angle should be standardized by means of external landmarks and measures should be taken through at least two complementary directions. From such data, the maximum and mean thickness of intima-media can be taken, as well as measurements of the lumen diameter. It is recommended that semi-automated edge detection software be used to identify the borders (3, 4).

Thorough guidelines on the use and measurement of CIMT have been published, including percentile CIMT data by sex, age and ethnicity (3) allowing for standardization of the method as well as reference ranges to be calculated for smaller studies. CIMT imaging has been validated against *in vitro* histology (5, 6).

Success rates for imaging the common carotid is >90%, in the bifurcation is 64–77%, and in the internal carotid 31–48% (7, 8). B-mode ultrasonography can more readily identify non-obstructive plaques than Doppler ultrasound, given that Doppler velocity does not increase significantly until >50% lumen obstruction is observed. However, it should be noted that while there is good agreement on the morphological evaluation of plaques, measurements of plaque thickness is subject to a higher incidence of measurement error (9).

### 3D ultrasound

Serial 2D ultrasound images can be computed to reconstruct the 3D volume. This requires specialized software and probes, but gives the advantages of reducing operator variability as well as allowing for the visualization of both the thickness and length of the plaque (10). 3D ultrasound is more sensitive to detect changes in plaque area (11).

### Pixel distribution analysis (PDA)

A limitation of CIMT scans is that no reliable characterization of plaque composition, and therefore stability, is available. Nevertheless, such techniques are under development and are currently available for research purposes. For example, it has been shown using PDA that the necrotic core of an unstable plaque is closer to the lumen and appears hypoechoic (12). PDA uses gray-scale image segmentation to map pixel brightness ranges across normalized longitudinal images. The result is a percentage composition of tissue composition in the plaque, including calcium, lipid and fibrous tissue. PDA can also provide information on the lipid core size and location (13).

### Contrast-enhanced ultrasonography

While most of the time US assessment of the carotid arteries is performed entirely non-invasively, image quality can be enhanced by the use of a contrast agent. For contrast-enhanced ultrasonography (CEUS), the contrast is typically microbubbles of an inert gas stabilized by a phospholipid shell (e.g. sulfur hexafluoride or octafluoropropane). For carotid CEUS, the carotid lumen and adventitia are enhanced, making luminal irregularities more readily detectable. Late-phase enhancement (6 min after contrast administration) suggests an increased inflammatory cell load within the plaque, representing a possible marker for early plaque rupture (14, 15). Careful evaluation of the patient medical history is needed before administration of contrast given the range of contraindications (16).

## X-ray based imaging

### Computed tomography angiography

**Figure 3:**
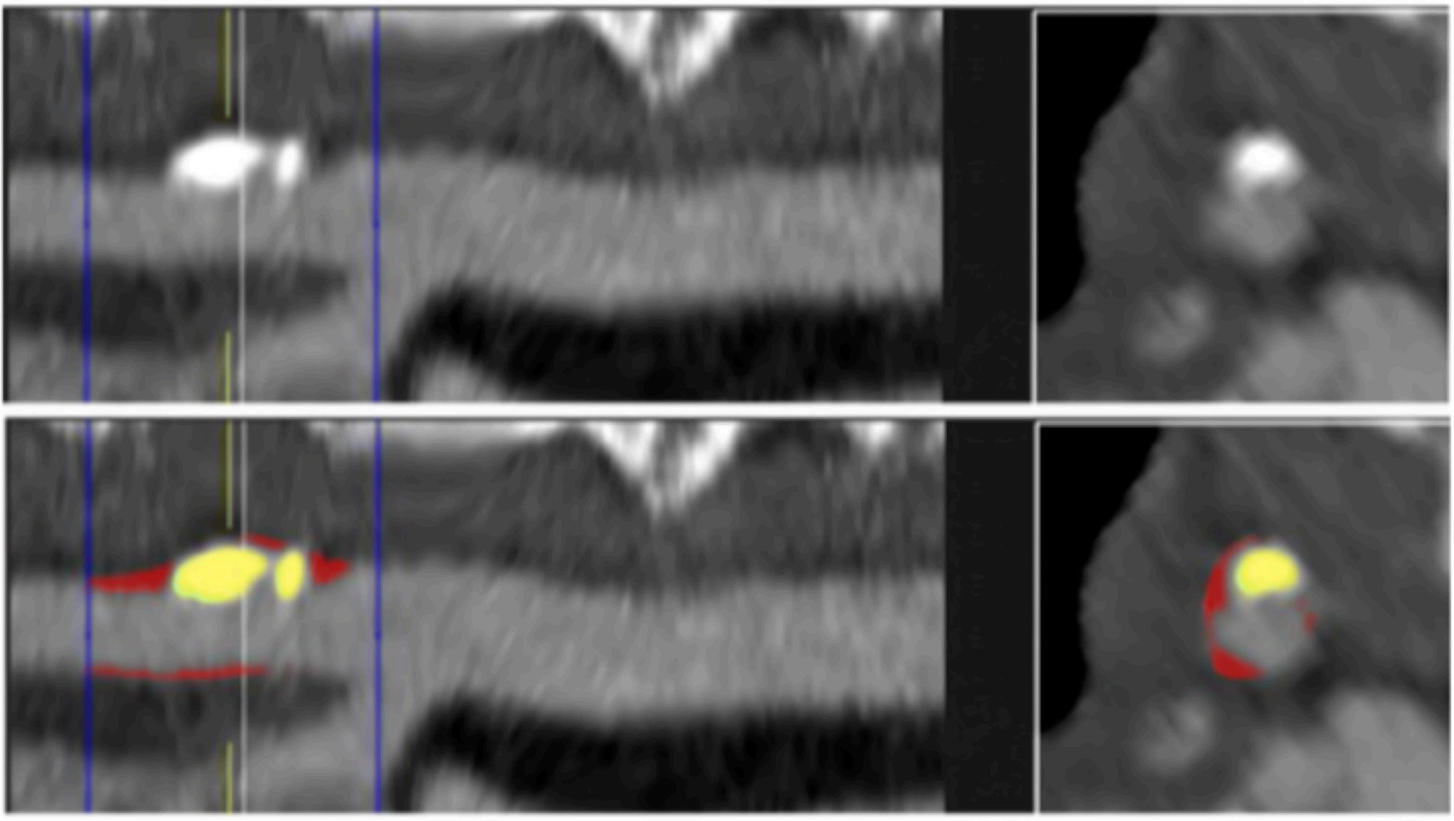
Example of plaque imaging by computed tomography angiogram in the common carotid artery with classification overlay to show non-calcified plaque (red) and calcified plaque (yellow). Reproduced from Ramanathan R, Dey D, Nørgaard BL, *et al.*; Carotid plaque composition by CT angiography in asymptomatic subjects: a head-to-head comparison to ultrasound; *European Radiology*, 2019 (34). Copyright 2019 John Wiley and Sons.

Computed tomography angiography (CTA) offers a fast acquisition (~10 s) imaging modality. With the advent of multi-detector row computed tomography (MDCT) the ability and quality of non-invasive angiograms has substantially increased; CTA has a spatial resolution of approximately 0.5–1 mm, but a relatively slow temporal resolution at 240–420 ms. However, newer dual-source CT (DSCT) scans may reduce the temporal resolution to ~65 ms, thereby making it near equivalent to that of magnetic resonance scans (17). Furthermore, DSCT allows for more accurate assessment of calcified plaque volume, as it uses two x-ray sources with different energies to achieve more detailed Hounsfield unit measurements (18). Plaques are typically imaged using bolus-tracking CTA. Calcification, lipid content and fibrous tissue are classified based on voxel Hounsfield units (19). However, densely calcified plaques may result in beam-hardening artifacts. Histopathological comparisons to DSCT show high agreement for the AHA classification of plaques, although it should be noted that type I and II lesions were seen only in histopathological analyses (18). Risks associated with radiation exposure and iodinated contrast administration should be taken into account before performing CTA (20, 21). An example of CTA imaging is shown in Fig. 3.

## Magnetic resonance-based imaging

### Magnetic resonance angiography

**Figure 4:**
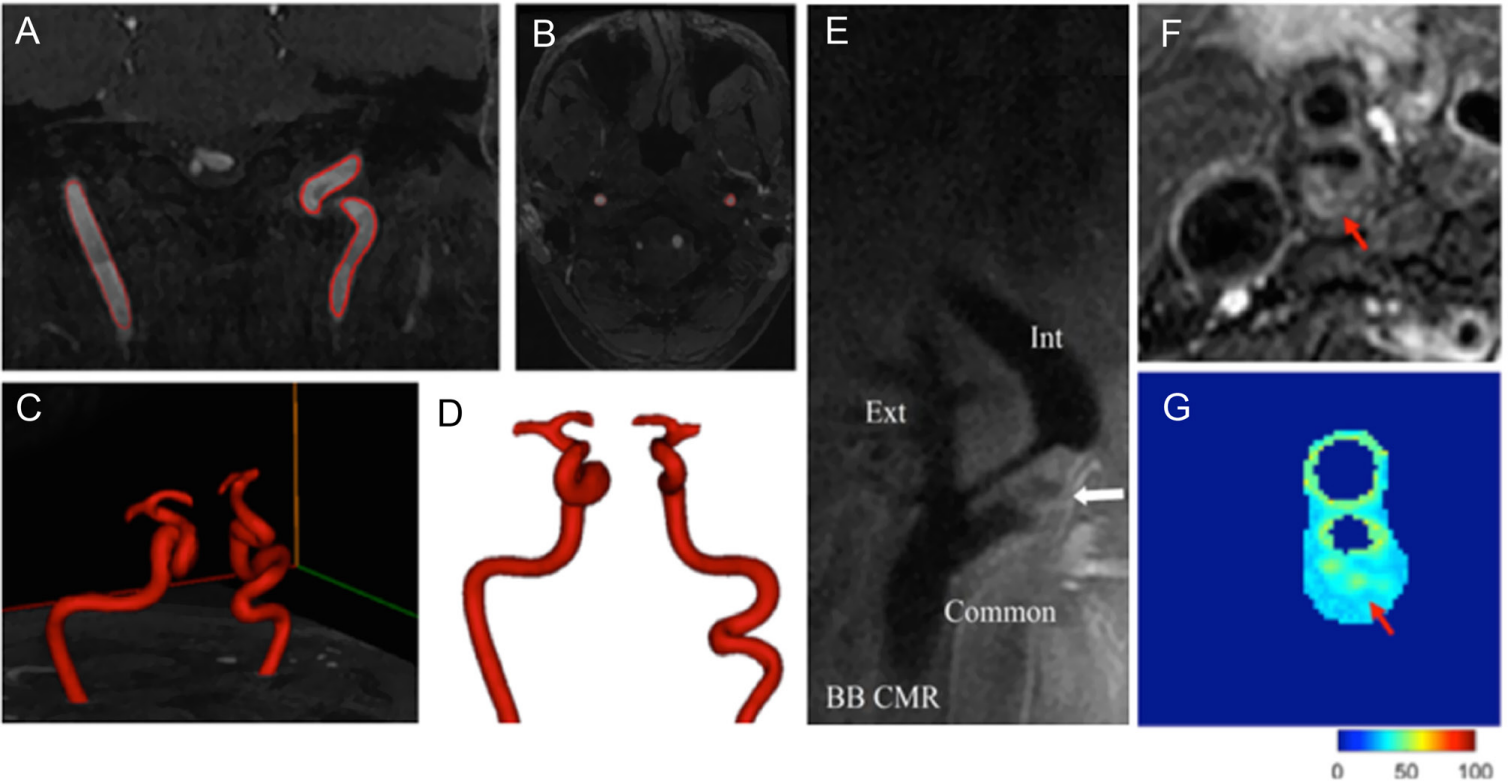
Example of segmentation of magnetic resonance angiography (MRA) data of the internal carotid artery (different views, A and B), including 3D reconstruction to reveal carotid anatomy (C and D). Example of black blood imaging in the internal carotid, the red arrow indicating a region of intraplaque hemorrhage (E) (reproduced from Yu *et al*. under the terms of the original Creative Commons CCBY Attribution License (35)). Example of T2 mapping of atherosclerotic carotid plaque, the red arrow indicating a region of intraplaque hemorrhage (F) (reproduced from Qi H, Sun J, Qiao H, *et al*.; Simultaneous T1 and T2 mapping of the carotid plaque (SIMPLE) with T2 and inversion recovery prepared 3D radial imaging; *Magnetic Resonance in Medicine*, 2018 volume **80**, pages 2598–2608 (36); copyright 2018 John Wiley and Sons), which is shown mapped in (G).

A range of MR techniques have been developed with specific technical advantages for imaging of different components of the plaque (22). Examples of different MR imaging techniques are shown in Fig. 4. Visualization of head and neck vessels including the carotid arteries in the research setting is typically performed using time-of-flight MRA, but other non-contrast MR imaging sequences may be of interest. MR imaging has the ability not only to quantify vessel lumen but also to characterize plaque composition including the necrotic core and calcification (23), fibrous cap (24) and inflammation (25). A commonly used research technique for plaque imaging is the double inversion recovery or ‘black-blood’ method. This uses a fast spin-echo sequence with double inversion recovery preparatory pulses resulting in a high contrast between the lumen and vessel wall. Newer sequences allow for the 3D acquisition so that the entire cervical carotid artery can be covered at a <1 mm^3^ resolution in less than 2 min (26). Moreover, fat suppression provides a clearer image and is essential for characterization of the plaque morphology. MRA can provide visualization of the vessel lumen, even when the vessel is highly calcified. However, the acquisition time is significantly longer than for CTA, and MRA has a relatively low spatial resolution (typically >1 mm). Nevertheless, MRA may be successfully used when CTA is contraindicated.

Recent advances in the application of T_2_ mapping techniques (27) have made high-resolution, non-contrast-enhanced plaque lipid quantification possible across the whole plaque area. The technique maps the T_2_ decay on a voxel-by-voxel basis, is validated against histological samples and has been shown able to distinguish recently symptomatic plaque with high sensitivity and specificity (28).

### Contrast-enhanced magnetic resonance angiography

Contrast-enhanced magnetic resonance angiography (CE-MRA) is a contrast-enhanced technique, typically using gadolinium or iron oxide-based contrast media (rather than iodine-based contrast used in CTA). Contrast MR may provide a clearer image of vessel morphology and plaques than non-contrast MR. To achieve this, calculations on the arrival time of the bolus is essential; imaging too early would yield an inadequate visualization of the vascular tree, whereas imaging too late may cause some contrast to spill into the venous system thereby adding noise to the anatomy under investigation (29). CE-MRA in the research setting may also be used to study preclinical and molecular imaging of the plaque. For a comprehensive review of CE-MRA see Makowski and Botnar (30).

## Other imaging techniques

### Positron emission tomography-based imaging

Positron emission tomography (PET) uses targeted radio-tagged molecular probes, which undergo beta-decay. While PET scans have traditionally suffered the same limitations as MRA, that is, long acquisition time and limited spatial resolution, newer hybrid PET-CT and PET-MR scanners have made PET imaging an option for studying plaques in further depth, combining the anatomical and/or metabolic images with specific markers, for example, for inflammation and hypoxia (31, 32).

## Summary

This mini review has briefly presented the main non-invasive imaging techniques to visualize the carotid plaque *in vivo*. Each technique has advantages and limitations and may be chosen depending on the availability, cost and clinical justification for its use. Common to all the imaging techniques presented here is the need for a skilled imaging professional to allow for high reliability and repeatability. While ultrasound-based imaging certainly is considered a first-line technique in clinical practice, the use of CTA or MRA needs to be considered in presence of significant stenosis with or without symptoms. MRA, CTA and PET are moving us toward a better understanding of the risk-stratification and pre-interventional monitoring of patients at risk of plaque rupture as well as early identification of plaque development.

## Declaration of interest

C B D is a consultant for Circle Cardiovascular Imaging (Calgary, Canada). The other authors have nothing to disclose.

## Funding

The authors acknowledge the support of the British Heart Foundationhttp://dx.doi.org/10.13039/501100000274 and the Wellcome Trusthttp://dx.doi.org/10.13039/100010269. C B D is partly funded by the National Institute for Health Research Biomedical Research Centrehttp://dx.doi.org/10.13039/501100012618 at University Hospitals Bristol NHS Foundation Trusthttp://dx.doi.org/10.13039/100012141 and the University of Bristol. The views expressed in this publication are those of the author(s) and not necessarily those of the NHS, the National Institute for Health Researchhttp://dx.doi.org/10.13039/501100000272 or the Department of Health and Social Care.
